# Evaluation of dentinal tubule penetration depth and push-out bond strength of AH 26, BioRoot RCS, and MTA Plus root canal sealers in presence or absence of smear layer

**DOI:** 10.15171/joddd.2018.046

**Published:** 2018-12-19

**Authors:** Sevinç Aktemur Türker, Emel Uzunoğlu, Nuhan Purali

**Affiliations:** ^1^Department of Endodontics, Faculty of Dentistry, Bülent Ecevit University, Zonguldak, Turkey; ^2^Department of Endodontics, Faculty of Dentistry, Hacettepe University, Sihhiye, Ankara, Turkey; ^3^Department of Biophysics, Faculty of Medicine, Hacettepe University, Sıhhiye, Ankara, Turkey

**Keywords:** Calcium silicate-based sealer, confocal laser microscopy, bond strength, penetration depth

## Abstract

***Background.*** This study compared the effect of smear layer on the penetration depth and push-out bond strength of various root canal sealers.

***Methods.*** A total of 90 extracted human mandibular premolars were assigned into 2 groups: smear layer preserved and smear layer removed. Then the roots were further divided into 3 subgroups according to the sealer tested: AH 26, BioRoot RCS and MTA Plus. Obturation was performed with gutta-percha and the relevant sealer was mixed with 0.1% rhodamine B. Three 1-mm-thick slices were obtained from the mid-third area of each root. Two slices were selected for the push-out test and the remaining slice was used to calculate the dentinal tubule penetration depth and percentage.

***Results.*** The retention of MTA Plus and BioRoot RCS was higher than that of AH 26 when the smear layer was preserved (P<0.05). BioRoot RCS showed the lowest penetration depth when the smear layer was removed (P<0.05).

***Conclusion.*** Dentinal tubule penetration of root canal sealers had a limited effect on their adhesion to root canal wall.

## Introduction

One of the main objectives of root canal treatment is to establish a fluid-tight seal to prevent contamination of the root canal system with bacteria. Gutta-percha with an endodontic sealer is the most widely accepted root canal filling material. Different types of endodontic sealers have been used in clinical practice, including zinc oxide, epoxy resin, silicone and methacrylate-based. Recently, a new class of root canal sealers, the calcium silicate-based, has been introduced. MTA Plus (Avalon Biomed Inc., Bradenton, FL, USA) is a powdered tricalcium and dicalcium silicate-based material that can be mixed with a liquid or a gel. It is used as a root canal sealer when the powder is mixed with gel.^1^ An advantage of MTA Plus is the smaller particle sizecompared to MTA.^2^ BioRoot RCS (Septodont, St.Maur-des-Fosses, France), a new calcium silicate-based root canal sealer which consists of powder and liquid, has been specifically developed for root canal filling. According to the manufacturer, the powder mainly consists of tricalcium silicate and the liquid is an aqueous solution of calcium chloride (curing accelerator) and excipients.^3^ Epoxy resin-based root canal sealers, such as AH26 (Dentsply DeTrey GmbH, Konstanz, Germany), have been commonly used for comparison because of its good physicochemical properties and adaptability to root canal walls.^4^

During root canal instrumentation, the smear layerforms on root canal walls. This layer is assumed to prevent the penetration of sealers into the dentinal tubules because it coats the dentin and blocks the orifice of the dentinal tubules. Therefore, it is assumed that the smear layer can affect the penetration depth and adaptation of root canal sealers.^5^

Dentinal tubule penetration depth is a performance measure of a root canal sealer. Previous studies have shown that the penetration of sealer into the dentinal tubules forms a physical barrier and entombs residual bacteria^6^ and improves retention of the root filling.^7^ However, no correlation has been found between sealer penetration into dentin tubules and sealability of the filling material.^8^

Several studies have focused on the dentin penetration of calcium silicate-based sealers.^9-12^ However,according to the authors’ knowledge no study has evaluated dentinal tubule penetration and retention of calcium silicate-based sealers with or withoutthe smear layer. Therefore, the aim of this study was to determine the effect of the smear layer on the adhesion and penetration depth of two calcium silicate-based sealers and the correlation between these two tests. The null hypothesis was that smear layer does not affect neither the push-out bond strength nor penetration depth of root canal sealers.

## Methods

Ninety single-rooted human mandibular premolar teeth were selected. The teeth were decoronated to achieve a standardize length of 16 mm. After determining the working length, 1 mm short of the apex, the root canals were instrumented with a series of ProTaper Universal file system (Dentsply, Maillefer, Ballaigues, Switzerland) to #40/06.

During instrumentation 2.5% NaOCl was used with a 27-gauge needle inserted to 1 mm short of the working length. The prepared roots were randomly assigned to two groups (n=45) as follows: the smear layer was preserved and the smear layer was removed by irrigation with 3 mL of 17% EDTA for one minute using a 27-gauge needle inserted to 1 mm short of the working length. To eliminate the EDTA action, irrigation was carried out with 3 mL of NaOCl followed by a final flush with 5 mL of distilled water and dried with paper points.

Then each major group was further assigned to three subgroups (n=15) according to the root canal sealer used: AH 26, BioRoot RCS and MTA Plus. All the sealers were mixed according to the manufacturers’ instructions and mixed with 0.1% rhodamine B dye (Sigma Aldrich Co., St Louis, MO, USA). The root canals were obturated with the relevant sealer in conjunction with an F4 single-cone gutta-percha. The samples were stored at 37°C for 7 days to set completely. Following the storage period, each root was sectioned horizontally to obtain three slices 1±0.1 mm in thickness from the mid-thirds.

### Push-out Bond Strength Test

The two slices of the mid-third area were selected for the push-out test in a universal testing machine (Instron Corporation, Canton, MA, USA) at a crosshead speed of 0.5 mm/min. Compressive force was applied to the obturation material through a cylindrical stainless-steel plunger (0.7 mm in diameter). The load applied at the time of displacement was recorded in Newton. The bond strength was calculated in MPa according to the formula:

Load/Adhesion surface area

The adhesion (bonding) surface area of each section was calculated as: [ (r_1_ + r_2_)_ /_ 2] x π x h, where π is the constant 3.14, r_1_ and r_2_ are the smaller and larger radii, respectively, and h is the thickness of the section in mm.

**Figure 1:**
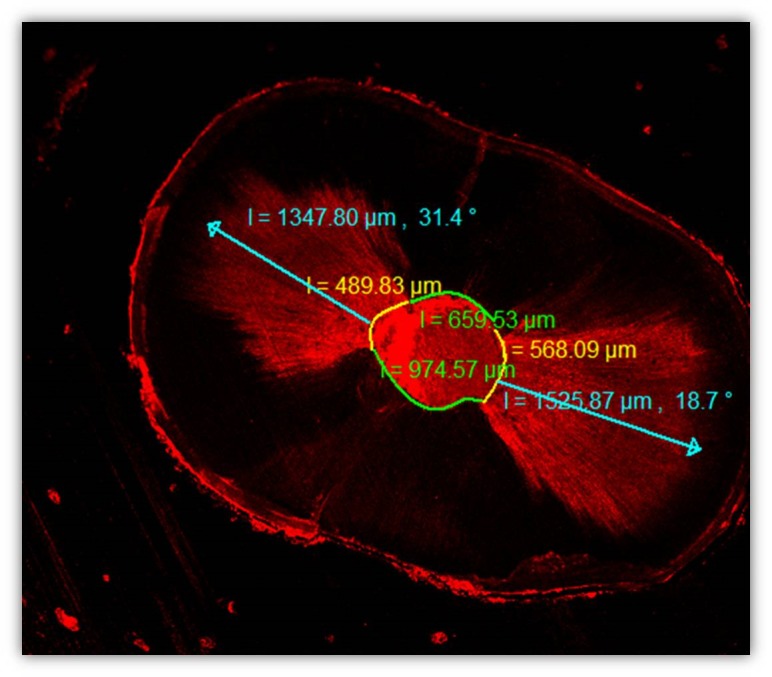
The measurements of dentin tubule penetration depth and percentage.

The slices were photographed under a CLSM (Zeiss LSM 510; Carl Zeiss, Jena, Germany) and a method of epifluorescence with wavelengths of absorption and emission for rhodamine B at 540/590 nm. The images were analyzed in the CLSM Image Browser (Carl Zeiss) to measure the longest penetration depth of the sealer and the percentage of the penetrated sealers into the dentinal tubules as shown in Figure 1.  The sealer penetration depths in the dentinal tubules were measured at their maximum depth for each specimen. The depth of penetration was measured from the canal wall to the point of maximum sealer penetration.^13^

### Statistical analysis

Data were analyzed using parametric tests (two-way ANOVA and post hoc Tukey test), considering the smear layer and root canal sealer as independent variables. Spearman’s correlation coefficient was used to assess the pairwise relationships between the two tests. The significance level was set at 5%.

## Results

### Bond strength to root canal dentin

According to the results, the push-out bond strength was significantly affected by the sealer type and smear layer removal/preservation (Table 1). The retention of calcium silicate-based sealers was higher than epoxy resin-based sealer when the smear layer was preserved (P<0.05). BioRoot RCS had higher retention compared to MTA Plus and AH 26 when the smear layer was removed (P<0.05). Removal of the smear layer decreased the retention of MTA Plus (P<0.05).

**Table 1:** Push-out bond strength values in absence or presence smear layer (mean ± SD, MPa)

**Groups**	**Smear Layer** ^+^	**Smear Layer** ^−^
**AH26**	1.53 ± 0.20^b^	1.53 ± 0.22^b^
**BioRoot RCS**	2.03 ± 0.47^a^	1.97 ± 0.46^a^
**MTA Plus**	2.02 ±0.55^a^	1.58 ± 0.41^b^

*Different letters are statistically significant (P<0.05)

### Confocal microscopy qualitative analysis

**Figure 2:**
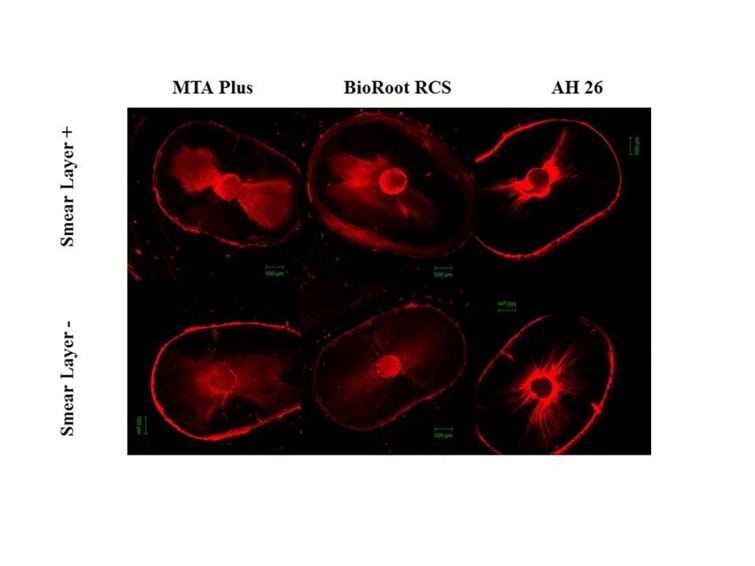
Representative images of each root canal sealer in the absence or presence of the smear layer.

Representative microscope views are showed in Figure 2. The smear layer did not affect the penetration depth of root canal sealers (P>0.05). However, the penetration depth of MTA Plus was significantly higher compared to BioRoot RCS and AH 26 when the smear layer was preserved (P<0.05). BioRoot RCS showed the lowest penetration depth when the smear layer was removed (P<0.05). Regarding the penetration percentage, there was no significant difference between the groups (P>0.05) (Table 2).

**Table 2:** Penetration depth (mm) and Percentage (%) of experimental groups (mean ± SD)

**Group**	**Penetration depth**		**Penetration Percentage**	
	**Smear Layer** ^+^	**Smear Layer** ^−^	**Smear Layer** ^+^	**Smear Layer** ^−^
**AH 26**	1413 ± 330^a^	1443 ± 100^ab^	73.00± 20.2^A^	78.50 ± 17.4^A^
**BioRoot RCS**	1248 ± 390^ac^	999 ± 360^c^	75.83± 27.1 ^A^	68.33 ± 28.0^A^
**MTA Plus**	1972 ± 330^b^	1701 ± 390^b^	75.67± 19.5^A^	88.08 ± 12.5^A^

*Different letters are statistically significant (P<0.05).

**Table 3:** Statistical analysis of the Spearman correlation used to assess the pairwise relationships between the tests

**Material**	**Relationship**	**Spearmen correlation**	**P value**
	**Bond strength-penetration depth**		
**AH 26**	Smear Layer ^+^	0.580*	0.048
	Smear Layer ^−^	0.490	0.880
**BioRoot RCS**	Smear Layer ^+^	0.406	0.191
	Smear Layer ^−^	0.636*	0.026
**MTA Plus**	Smear Layer ^+^	-0.259	0.417
	Smear Layer ^−^	-0.538	0.710

*Correlation is significant at the 0.05 level

Spearman’s correlation coefficient showed weak relation between the two tests. These relationships were not significant as P-values exceed the 0.05 level of significance, except AH 26/smear layer+ and BioRoot RCS/smear layer^−^. For MTA Plus with or without the smear layer, the relationship between the bond strength-penetration depths had a negative tendency.

## Discussion

Two measures of a sealer’s performance are its ability to penetrate into the dentinal tubules and adhere to root canal wall. Many factors might influence the penetration depth and retention of a root canal sealer to root canal wall. These factors include the physicochemical featuresof the sealer, smear layer, root canal morphology and obturation method. The present study focused on the effect of the smear layer on sealer penetration depth and bond strength of two calcium silicate-based sealers.

The penetration of root canal sealers into dentinal tubules decreases the interface between the core material and dentin, and retention of the core material might be improved by mechanical interlocking. The push-out bond strength test provides valuable information about retention of sealers on root canal walls.^14^ Horizontal root sections from the mid-root area of teeth were used for bond strength and analysis of penetration depth and percentage under CLSM. This was carried out to standardize the samples.

The results of the present study showed significantly different performance amongst the tested materials in the absence or presence of the smear layer. The null hypothesis was partially refuted. In terms of bond strength, the smear layer preservation resulted in significantly better results for MTA Plus, but this result was not observed for AH26 and BioRoot RCS, whose results were similar to those of the smear layer removal group. On the other hand, in terms of penetration depth and percentage of root canal sealers no significant differences were found in the absence or presence of the smear layer.

According to the results of this in vitro study, regardless of the smear layer the bond strengths of calcium silicate sealers were higher than epoxy resin-based sealers. It was reported that there is a chemical bond with a micromechanical locking via cement tags in the dentinal tubule,which is referred to as the mineral infiltration zone, between calcium silicate cements and the dentin surface.^15^ It can be speculated that because of this interaction of calcium silicate-based sealer with the dentin surface, their adhesion to the root canal wall is better than that of epoxy resin-based sealer, which bonds to root canal wall via covalent bonds.

According to the results of the push-out bond strength test, when the smear layer was preserved calcium silicate-based sealers exhibited higher bond strength values compared to epoxy resin-based sealers. However, in the absence of the smear layer AH26 had similar bond strength compared to MTA Plus.When root canal sealers were evaluated in their own right, it was found that the smear layer improved adhesion of MTA Plus to root canal wall. On the other hand, smear layer removal had no effect on the adhesion of neither BioRoot RCS nor AH 26. It can be concluded that the smear layer has an important role in the formation of the interfacial layer between the MTA Plus and root dentin. Yildirim et al^16^ reported that due to the moisture condition of the root canal wall, the smear layer, which acts a coupling agent between dentin and MTA, might have a positive effect on the adhesion of MTA to the root canal wall. Regarding this issue the literature presents conflicting results. In a previous study it was shown that smear layer removal adversely affected the adhesion between calcium silicate cements and dentin;^17^ in the same study, AH Plus showed similar adhesion in the absence/presence of the smear layer, consistent with the results of the present study.^18^ Similarly, Shokouhinejad et al^18^ showed that the smear layer did not affect the bond strength of an epoxy resin-based (AH Plus) and a calcium silicate-based sealer (EndoSequence BC).

In terms of dentinal tubule penetration depth, it was found that the smear layer had no effect on penetration depth and percentage of any root canal sealer. The results found for sealer penetration depth of AH26 are in agreement with a previous research by Kuci et al.^19^ Their study showed that absence or presence of the smear layer had no effect on the penetration depth of AH26. However, when the smear layer was preserved MTA Plus showed deeper penetration than BioRoot RCS and AH26. MTA Plus has similar contents compared to white MTA (Angelus), but with fine particle size and high specific surface area of the powder.^2^ It can be concluded that these particle sizes of MTA Plus might be well suited for better penetration into the dentinal tubules. On the other hand, when the smear layer was removed penetration depth of BioRoot RCS was less than that of AH26 and MTA Plus. This might have been caused by the relatively higher fluidity of AH 26 when it contacted the exposed dentin tubules when the smear layer was removed.

The effect of the smear layer on the bond strength and penetration depth of these two calcium silicate-based sealers used in present study, to the best of our knowledge, has not been investigated yet. Therefore, comparison was not possible due to the different types of sealers used in previous studies. Further investigations are required to assess the effect of the smear layer on behavior of MTA Plus and BioRoot RCS.

## Conclusion

Within the limitations of this *in vitro* study, it can be concluded that smear layer removal adversely affects the adhesion of MTA Plus; however, the same did not hold for AH26 and BioRoot RCS. Additionally, smear layer removal or preservation did not affect the penetration depth and percentage of any root canal sealer. Dentinal tubule penetration had limited effect on the push-out bond strength of the root canal sealers.

## Acknowledgments

The authors are grateful to Professor Nuhan Purali for his valuable support for the confocal microscopy analysis.

## Authors’ contributions

SAT and EU designed the study, and SAT carried out the experiments and data analysis. SAT and EU wrote and edited the manuscript. Both authors read and approved the final manuscript.

## Funding

This study did not receive a funding.

## Competing interests

The authors declare no competing interests with regards to the authorship and/or publication of this article.

## Ethics approval

Teeth used in this study were selected from a collection of teeth that had been extracted for reasons unrelated to this study.
